# Saline Accelerates Oxime Reaction with Aldehyde and Keto Substrates at Physiological pH

**DOI:** 10.1038/s41598-018-20735-0

**Published:** 2018-02-01

**Authors:** Shujiang Wang, Ganesh N. Nawale, Sandeep Kadekar, Oommen P. Oommen, Naresh K. Jena, Sudip Chakraborty, Jöns Hilborn, Oommen P. Varghese

**Affiliations:** 1 Polymer Chemistry Division, Department of Chemistry, Ångström Laboratory, Uppsala University, Box 538, 751 21 Uppsala, Sweden; 2 Bioengineering and Nanomedicine Lab, Faculty of Biomedical Sciences and Engineering, Tampere University of Technology, and BioMediTech Institute, Tampere, 33720 Finland; 3 Condensed Matter Theory, Materials Theory Division, Department of Physics and Astronomy, Ångström Laboratory, Uppsala University, Box 516, 751 21 Uppsala, Sweden

## Abstract

We have discovered a simple and versatile reaction condition for oxime mediated bioconjugation reaction that could be adapted for both aldehyde and keto substrates. We found that saline accelerated the oxime kinetics in a concentration-dependent manner under physiological conditions. The reaction mechanism is validated by computational studies, and the versatility of the reaction is demonstrated by cell-surface labeling experiments. Saline offers an efficient and non-toxic catalytic option for performing the bioorthogonal-coupling reaction of biomolecules at the physiological pH. This saline mediated bioconjugation reaction represents the most biofriendly, mild and versatile approach for conjugating sensitive biomolecules and does not require any extensive purification step.

## Introduction

**Figure 1:**
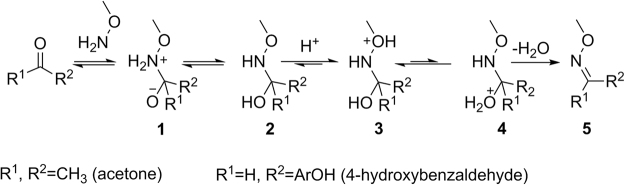
Reaction pathway for oxime formation.

Oxime chemistry is one of the most proficient coupling strategies adopted for bioconjugation of molecules, owing to its simplicity and efficiency^1–3^. It displays very high chemoselectivity and hydrolytic stability, making it a reaction of choice for biologists as an alternative to ‘click-chemistry’^4^. However, it suffers from major disadvantages such as slow reaction rate at physiological pH^5^, poor reactivity with keto substrates and the requirement of high substrate concentrations that significantly limit its potential applications^6–8^. Therefore, identifying biocompatible catalysts that can accelerate oxime formation kinetics (even for keto-substrates) at physiological pH is highly desirable. The rate-limiting step of oxime formation at neutral pH is the dehydration step, which is difficult due to the α-effect of aminooxy oxygen heteroatom (Fig. 1)^9^. Due to the α-effect, the protonation of ether oxygen (OR) is favoured over the hydroxyl (OH) one to get intermediate **3**, while the formation of key intermediate **4**, required for generating oxime product, is not preferred (Fig. 1). This insurmountable challenge has prompted several groups to develop organic catalysts that increase the reaction kinetics by forming a Schiff base with the catalyst that subsequently underwent a sequential transimination reaction with the incoming aminooxy nucleophile^7,8,10^. The most common nucleophilic catalyst is aniline that was first demonstrated by Jencks^5^ and later by Dawson^8^. However, aniline is not very efficient in promoting oxime reaction at physiological pH. Recently, more reactive nucleophilic catalysts such as 2-(aminomethyl)benzimidazole^11^ and anthranilic acid^12^ have been reported to improve the reaction kinetic at neutral pH. The major disadvantage with these organic catalysts is that they have poor solubility under aqueous condition. The solubility problem is circumvented by derivatizing it with hydrophilic groups, though that also alters its *pKa* and the nucleophilic character^13^. These catalysts, in general, are less efficient with keto substrates owing to their poor electrophilicity as compared to the aldehyde substrates^3,12^. Another challenge with organic catalysts is the need for purification: the organic catalysts need to be removed after accomplishing the bioconjugation reactions, as they may interfere with the biological activity or induce undesired toxicity^3^. Therefore, identifying a non-toxic, biocompatible alternative is of paramount importance.

Although most of the strategies include nucleophilic catalysts, there have been a few reports of non-nucleophilic catalysts that can accelerate oxime reaction rate. This includes *ortho*-boronic acid-based aldehyde and keto substrates that enhance both forward and backward reactions at physiological pH^14^. We have recently shown that organic carboxylates could promote oxime formation at physiological pH with both aldehyde and ketone substrates by stabilizing the key reaction intermediates through hydrogen bond formation^15^.

It is noteworthy to mention that all the catalysts reported in the literature that were developed to promote oxime reaction under physiological conditions used a buffer with different salt concentrations. The influence of salts in these reactions was generally overlooked. Here, we show for the first time that aqueous NaCl solution is capable of promoting the oxime reactions at physiological pH in a concentration-dependent manner. This effect is attributed to the stabilization of the charged transition states that favour the key rate-limiting elimination (dehydration) step. Our observation is supported by previous reports that demonstrated the use of aqueous NaCl to stabilize the charged transition state, and catalyse reactions such as Michael addition^16^ and Diels–Alder reactions^17^.

## Results and Discussion

### Oxime reaction with small molecule substrates

**Figure 2:**
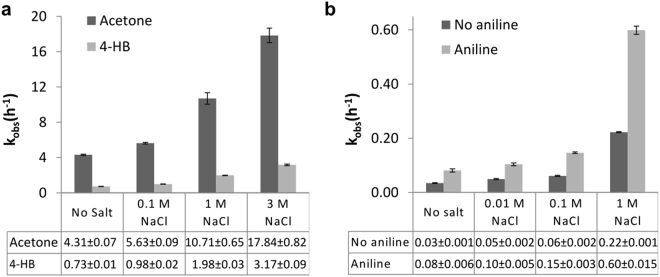
Comparative pseudo-first-order oxime reaction kinetics analyzed by ^1^H NMR and UV-Vis spectrometry. (**a**) The reaction of (aminooxy)methane and acetone/4-hydroxybenzaldehyde (4-HB) using different concentrations of NaCl in phosphate buffer (PB) as analysed by ^1^H NMR. (**b**) Oxime formation kinetics of (aminooxy)methane and 4-nitrobenzaldehyde using a different concentration of NaCl in PB containing 10% N,N-dimethylformamide as determined by UV-Vis spectroscopy. ± Therefore, the pseudo-first-order reaction was performed with 32 µM of 4-nitrobenzaldehyde (a chromogenic substrate) and 1 mM of (aminooxy)methane in phosphate buffer (PB), with different concentrations of NaCl (Fig. 2b).

To determine the effect of saline on oxime formation kinetics, we first performed a small molecule pseudo-first-order model reaction in 0.01 M deuterated phosphate buffer (dPB, pD 7.4, with different concentrations of NaCl) at 25 °C. Water-soluble 4-hydroxybenzaldehyde and acetone were used as model electrophiles, and (aminooxy)methane (NH_2_OCH_3_) was used as the model nucleophile. The reactions reached 100% conversion, and the rate of condensation reaction was monitored using ^1^H NMR (Fig. S1). Interestingly, when 100 mM NaCl was used in the reaction mixture, we observed a ~1.3-fold increase in the reaction rate with both the carbonyl substrates, as compared to that of the uncatalysed reaction (Fig. 2a). However, when the salt concentration was further increased to 1 M and 3 M, we observed a gradual increase in reaction rate (Fig. 2a). Specifically, with 3 M NaCl, the rate of reaction increased ~4-fold with both substrates. Further increase of the salt concentration resulted in partial precipitation of the product (salting-out effect) and, therefore, ^1^H NMR analysis could not be performed.

Since ^1^H NMR experiments require high substrate concentrations (in mM range) that are generally accepted to yield oxime product at a reasonable reaction rate^18^, we decided to estimate the reaction kinetics at significantly lower concentrations (μM range) and physiological pH by UV based methods.

The reaction kinetics was followed by UV-Vis spectrophotometer. When we used 10 mM NaCl in the reaction mixture, a ~1.7-fold enhancement in reaction rate was observed. Gratifyingly, when the NaCl concentration was further increased to 100 mM and 1 M, a ~2.0-fold, and ~7.3-fold increase in the reaction rate was observed, respectively (Fig. 2b). Interestingly, when 1 M NaCl and 1 mM aniline were used concomitantly, the reaction rate increased by ~20-fold (Fig. 2b). In general, these results revealed that saline could indeed accelerate oxime formation with both aldehyde and keto substrates at physiological pH.

### Computational analysis

**Figure 3:**
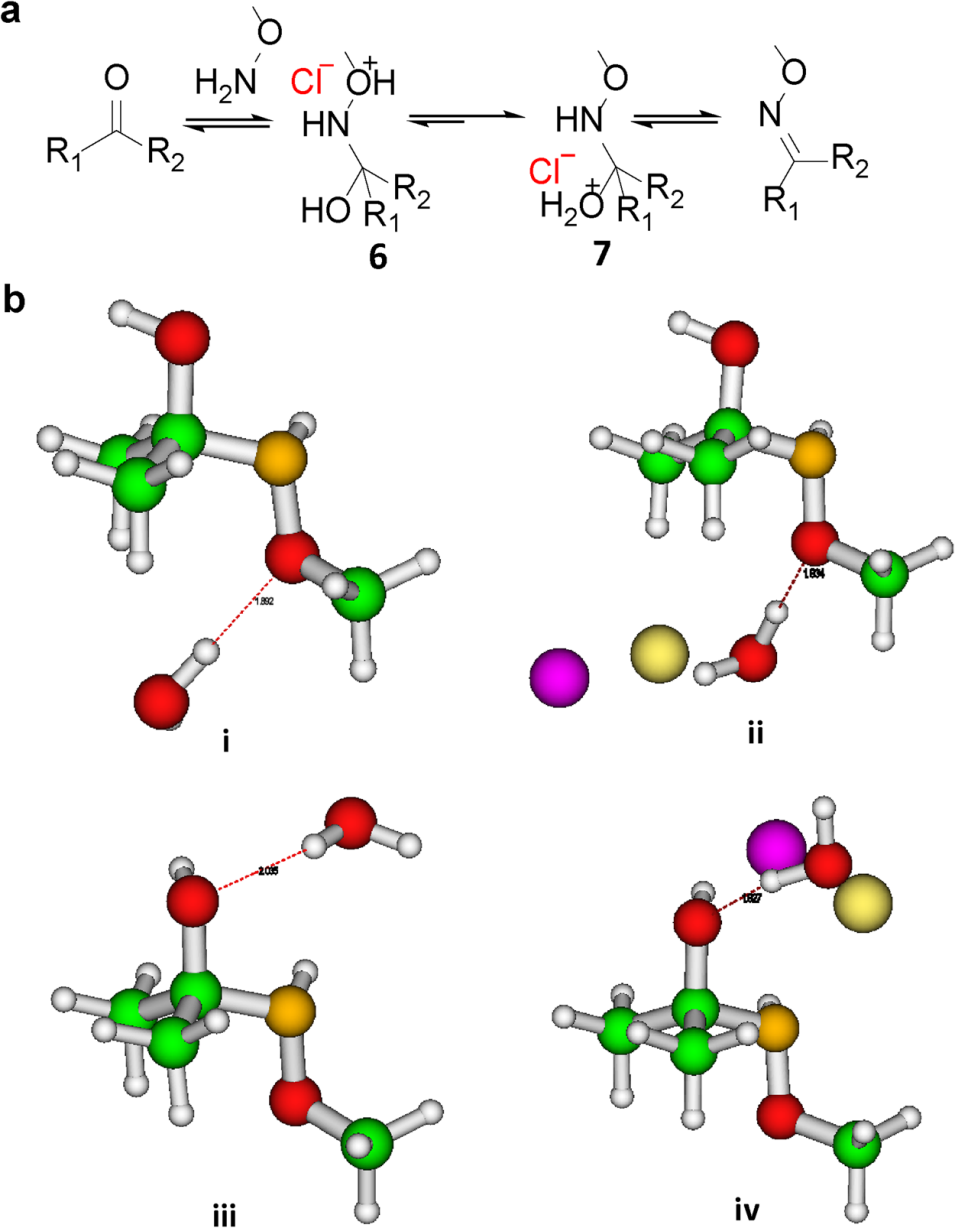
The proposed mechanism of salt-catalysed oxime formation. (**a**) Reaction scheme of salt-stabilized transition states. (**b**) DFT optimized (B3LYP/6–31 G(d) level of theory) geometries of the molecule showing two possible O-protonation sites (-OR and -OH) in the presence (ii and iv; which are equivalent to **6** and **7** in Fig. 3(a) respectively) and absence (i and iii) of NaCl. The dotted lines indicate possible hydrogen bonding involving protonated O with H of H_2_O. The computed charges on protonated O atom (–OR/–OH) are −0.47, −0.48, −0.77, −0.81 in units of e-, respectively for (i–iv). (Atom Colors: O (Red), H (White), N (Dark Yellow), C (Green), Na (Light Yellow), Cl (Purple).

We believe, this increase in reaction rate was due to the stabilization of the charged transition state^19^ as well as an increase in the local concentration of the organic reactants at high salt conditions (salting out effect). To decipher the role of saline in stabilizing the charged transition state and driving the forward reaction, computational studies were performed on the hemiaminal intermediates **6** and **7** in the presence and absence of NaCl (Fig. 3a). It was anticipated that the α-effect of aminooxy derivative would favour protonation of the ether oxygen (–OR; **6**) over hydroxyl oxygen (–OH; **7**) (Fig. 3a). To estimate the influence of α-effect on the propensity of protonation at a particular oxygen site (**6** or **7**), electronic structure calculations were performed using the Density Functional Theory (DFT) formalism. We considered the protonation of O-site by placing H_2_O in its vicinity and the hydrogen bonding of protonated O-site with H (of H_2_O) was shown as dotted lines in Fig. 3(b). The energetics of the two protonated sites (**6** and **7**) based on DFT calculations demonstrated that the difference in total energy between the two intermediates is not significant and –OH protonated structure 7 had a slight preference over the –OR counterpart **6**, with an energy difference of 2.4 kcal/mol (Fig. 3b). The introduction of NaCl in both the systems, however, dramatically changed the electronic structures of the two intermediates. With the addition of NaCl, the energy difference between the two-protonated species was found to be 20.8 kcal/mol with intermediate **7** significantly more stable (~9-fold) than **6**.

To achieve a better understanding of the protonation process and the charge transfer, the charge accumulation on the oxygen atom of the respective protonated site was analysed. Calculation of electrostatic potential (ESP) representing the electropositive and electronegative sites of a molecule is useful to show which region of the molecule is charge deficient and which is charge sufficient. In this research, we did not thoroughly analyze the charge difference of the full molecule (**6** and **7**, in presence or absence of NaCl) because the changes are really small to give any observable effect. Instead, we focused on relative affinities of protonated O-sites on the basis of charges, in the absence and presence of NaCl. In the case of 7, the charge on the hydroxyl oxygen atom was −0.77e- in the absence of NaCl, which changed to −0.81e- when NaCl was introduced into the system. This sizeable charge difference coupled with the higher magnitude of negative charge expedited the protonation of 7 due to ‘salt-effect,’ which led to the stabilization of the rate-limiting transition state. This observation was consistent with the energetics of the two charged transition states, as discussed previously.

On the other hand, the charge accumulated on the ether oxygen atom of **6** in the absence or presence of NaCl was −0.47e- and −0.48e- respectively, where the change was almost negligible, and the total charge was also less compared to the former case. To present a simpler electrostatics picture, the oxygen atom has a lower negative charge, and consequently, has a smaller propensity for protonation. In these experiments, another favourable stabilizing interaction was observed between the hydrogen atom of the hydroxyl group in **7** with the chloride ion of NaCl. This interaction enhanced the overall negative charge character of the adjacent hydroxyl oxygen atom and made it more favourable for protonation (Fig. 3b). This stabilizing factor (H…Cl interaction) was absent in the case of **6**. Overall, the computational analysis clearly indicates that the protonation of intermediate **7** is more favourable over **6** in the presence of NaCl, which is in good agreement with the experimental observation of accelerated oxime formation kinetics in the presence of NaCl.

### Oxime ligation on the cellular surface

**Figure 4:**
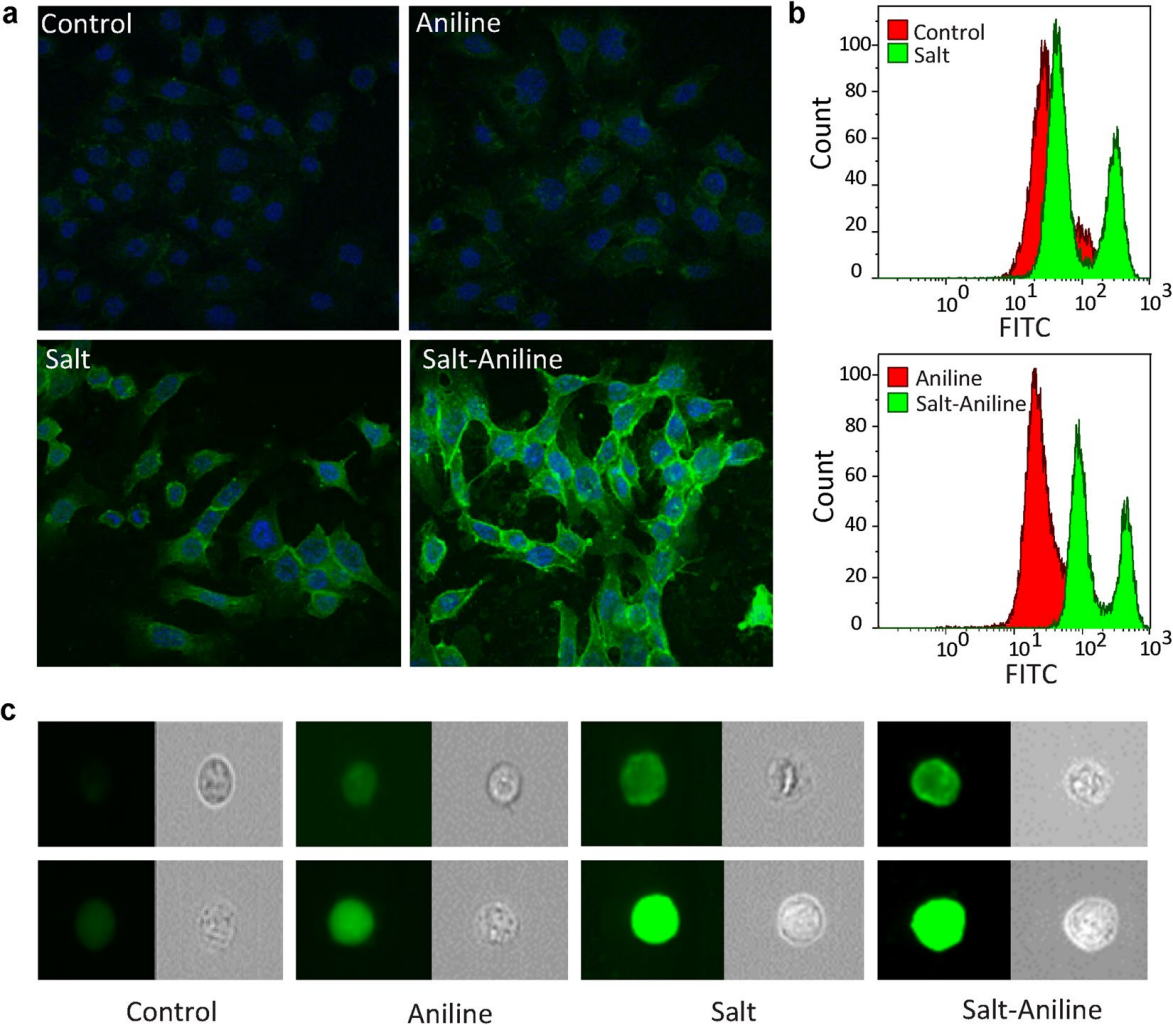
Oxime labelling of periodate treated HCT116 cells as analysed by fluorescence microscopy and flow cytometry using aminooxy-FITC as a nucleophile with different catalysts namely, PB as control, aniline, NaCl and NaCl having aniline as co-catalyst. (**a**) Confocal microscopy of HCT116 cells is indicating oxime labelling efficiency with different catalysts. All cells were fixed with 4% formaldehyde, and the nucleus was stained with DAPI after oxime reaction. (**b**) Amnis FlowSight® flow cytometry analysis of FITC-labelled HCT116 cells with different catalysts as mentioned above. (**c**) Fluorescent and phase-contrast (grayscale) images for labelling as analysed by Amnis FlowSight® flow cytometer. Top lane - Cell surface labelling. Bottom lane - cell surface labelling and intracellular trafficking of the fluorophore.

Finally, we validated the salt mediated oxime reaction by performing bioorthogonal ligation on the cell-surface of mammalian cells. We performed the oxime-based bioorthogonal ligation to modify cell surface glycoproteins, in the presence of aniline or NaCl as catalysts. For this purpose, an aldehyde functional group was generated on human colon cancer cell line (HCT116 cells) by periodate oxidation of cell-surface sialic acid residues using reported procedure^20^. To monitor the oxime reaction directly on the cell surface, a fluorescein-functionalized aminooxy derivative was used and live cell-surface labelling experiment was performed in PB (pH 7.4) with aniline, aqueous NaCl or a combination of both as catalysts (Fig. 4a).

Surprisingly, confocal microscopy of these labelled cells revealed that aniline (in the absence of NaCl) did not promote significant cell surface labelling (Fig. 4a). Although aniline promoted oxime formation with small molecules, the dense extracellular matrix in live cells can pose a challenge to perform reactions, presumably resulting in less cell surface labelling. In fact, aniline mediated labelling (without NaCl) was inferior to the labelling observed in the PBS (PB with NaCl) system. However, efficient labelling was observed in the PB group containing aniline-NaCl combination. This clearly indicates that the salts present in the buffer play an important role in such bioconjugation reactions. The catalytic activity previously reported for aniline was unintentionally augmented by the buffers used in the experiments^20^. We further probed the bioconjugation reaction efficiency on cell-surface by performing fluorescence-activated cell sorting (FACS) experiments (Fig. 4b). Corroborating with the confocal microscopic analysis (Fig. 4a), we did not observe any cell surface labelling in the control group without any catalysts (Fig. 4b). On the contrary, both PBS and aniline-PB with NaCl showed significant labelling which displayed two distinct populations of labelled cells. To better understand the effect of covalent conjugation reaction on the cell surface, FACS analysis was carried out using Amnis® imaging flow cytometer that combines cell sorting with high-resolution microscopy^21^. We believe the extracellular labelling of cells was due to fast reaction kinetics as NaCl or NaCl with aniline catalyzes the reaction, which resulted in the first peak. If the reaction kinetics is too slow, small hydrophobic molecules diffuse through the cell membrane which results in intracellular localization of the fluorophore that resulted for the second peak.

These experiments revealed that PBS group and PB with aniline-NaCl showed efficient cell surface labelling (Fig. 4c, top lane) with some intracellular localization of the conjugated fluorophore (Fig. 4c, bottom lane). The control groups (PB and PB with aniline) on the other hand, did not show significant cell surface labelling but instead displayed some intracellular trafficking of the fluorophore (Fig. 4c, bottom lane and Fig. S5, Supplementary Information). Of note, the cells that did not have any cell-surface aldehyde groups (i.e., cells without periodate treatment) did not show any intracellular localization of fluorophore or extracellular labelling of cells (data not shown). These experiments clearly indicate that salt indeed influences oxime reaction kinetics on cell surfaces at physiological pH.

## Conclusions

In summary, we present a simple strategy to modulate the oxime formation kinetics at physiological pH by changing the salt concentrations. Saline-promoted oxime formation was efficient with both the aldehyde and keto substrate, even at low substrate concentrations. Increasing the NaCl concentration or using NaCl in combination with other organic catalysts such as aniline could further enhance the reaction rates for such reactions. The computational analysis further corroborated the reaction mechanism, which indicated that the addition of NaCl stabilized the key reaction intermediate favouring the rate-limiting dehydration step without using any organic catalysts. Interestingly, the cell surface labelling experiment clearly demonstrated the role of the salt in performing labelling. However, no cell surface labelling occurred when aniline was used as a catalyst; albeit in the presence of NaCl. This simple salt modulated oxime formation strategy offers a great advantage for bioconjugation application, particularly in the case of sensitive water-soluble biomolecules such as cytokines and antibodies.

## Methods

### Materials and reagents

All reagents, including (aminooxy)methane, acetone, acetaldehyde, 4-hydroxybenzaldehyde, 4-nitrobenzaldehyde, sodium chloride, aniline, sodium periodate, formaldehyde, and glacial acetic acid, were purchased from Sigma–Aldrich (Sweden). Aminooxy-FITC was purchased from Thermo Fisher Scientific (Sweden). The ^1^H NMR experiments were carried out on Jeol JNM-ECP Series FT-NMR system at a magnetic field strength of 9.4T, operating at 400 MHz. Lambda 35 UV/Vis spectrophotometer from PerkinElmer instruments was used for spectroscopic analysis.

### Pseudo-first-order oxime formation kinetics analyzed by ^1^H NMR

All the reagents used in oxime ligation kinetics were dissolved in dPB (10 mM, pD 7.4, pD = pH + 0.4). The pseudo-first-order reactions were performed using a 30-fold excess of aminooxy (30 mM) with respect to aldehyde/keto derivative (1 mM) in dPB. ^1^H NMR spectra were recorded at desired time points, and the extent of oxime ligation was quantified by comparing the peak integral of carbonyl substrate and the peak of oxime product (Fig. S1, Supplementary Information). The pseudo-first-order reaction rate was calculated using equation S1–S3. Representative examples of these pseudo-first-order reaction rate are shown in figure S2. Comparative pseudo-first-order rate kinetics have been plotted in Fig. 2a.

### Pseudo-first-order oxime formation kinetics analyzed by UV-Vis

Samples were mixed in a 3 ml capacity quartz cuvette having path length 1 cm. PB (10 mM, pH 7.4) containing catalysts and 10% (v/v) DMF was used as a reference. 3 µl of 4-nitrobenzaldehyde stock solution (32 mM in DMF) was added to 2.982 ml of the above mentioned mixture, and UV-Vis absorbance (from 250 nm to 400 nm) were recorded. The reaction was initiated by addition of 15 µl (aminooxy)methane stock solution (200 mM in 10 mM PB, neutralized to pH 7) and absorbance was recorded at specific time intervals. Time-dependent UV/Vis spectral changes are shown in figure S3. Absorbance at the 307 nm was plotted against time. Pseudo first order reaction rate was calculated using equation (S4–S6). Representative examples of these time-dependent single-wavelength absorbance changes can be found in figure S4. Pseudo-first order rate kinetics was plotted in Fig. 2b.

### Cell culture experiments

HCT116 cells were cultured in Dulbecco’s Modified Eagle Medium (DMEM), supplemented with 10% fetal bovine serum (FBS) (Gibico) and 1% antibiotics (10.000 U penicillin and 10 mg/ml streptomycin, Sigma). Cells were maintained in a humidified incubator at 37 °C and 5% CO_2_.

### Fluorescence microscopy

HCT116 cells were seeded at a density of 50000 cells per well in a slide well for 24 hours before treatment. Cells were treated with 1 mM NaIO_4_ in PBS for 30 minutes at 4 °C, washed with PB and treated with 50 μM aminooxy-FITC. The oxime reaction was performed using different catalysts namely, 3 M NaCl; 1 mM aniline; and 1 mM aniline together with 3 M NaCl in PB (pH 7.4) for 90 minutes at room temperature. PB without NaCl was used as a control. After the reaction, cells were fixed with 4% formaldehyde for 20 minutes, washed with PBS and stained with DAPI for 5 minutes. Cells were then imaged using Carl Zeiss LSM 710 confocal microscope with 67X objective.

### Flow cytometry analysis

To perform flow cytometry analysis, 10,000,000 HCT116 cells were trypsinized and treated with 1 mM NaIO_4_ in PBS for 30 minutes at 4 °C in suspension. Thereafter, 2,000,000 cells were washed with PB and treated with 50 μM aminooxy-FITC. The oxime reaction was performed using different catalysts namely, 3 M NaCl; 1 mM aniline; and 1 mM aniline together with 3 M NaCl in PB (pH 7.4) for 90 minutes at room temperature. PB without NaCl was used as a control. Cells were then washed and resuspended in PBS. Further samples were analysed by flow cytometer using Amnis FlowSight®. 5000 individual cells for each treatment were imaged while being analyzed by the instrument. IDEAS software was used to analyse the data.

### Computational analysis

In order to envisage the protonation possibility at which particular O-sites out of –OH and –OR, electronic structure calculations based on Density Functional Theory (DFT) formalism were carried out to mimic the experimental investigation. The organic molecules were originally modelled by Gaussview program and subjected to geometry relaxation. Then, these model systems had been fetched by adding –H and –OH at the respective –OH and –OR sites and subsequent geometry relaxation were performed of both the systems using 6–31 G(d) basis sets using Gaussian09 program^22^. For the sake of accuracy, hybrid exchange correlation functional B3LYP was used throughout the work for full geometry optimization. The salt effect modulation in the experiment was modelled by introducing NaCl near the vicinity of the protonation sites. The charge accumulation on –O site during the protonation on –OH and –OR site with and without NaCl is worth to estimate in this modelling and it had been determined using Natural Population Analysis (NPA) as embedded in the code.

## Electronic supplementary material

Supplementary Information
