# Supplementary material for: Saline Accelerates Oxime Reaction with Aldehyde and Keto Substrates at Physiological pH
Source: Sci Rep. 2018 Feb 1;8:2193. doi: 10.1038/s41598-018-20735-0 (PMC5794741; doi:10.1038/s41598-018-20735-0)
Supplement: Supplementary file 1 — Supplementary Information [file 41598_2018_20735_MOESM1_ESM.docx]

Supplementary Information

Saline Accelerates Oxime Reaction with Aldehyde and Keto Substrates at Physiological pH

**Shujiang Wang, ^a^ Ganesh N. Nawale, ^a^ Sandeep Kadekar, ^a^ Oommen P. Oommen, ^b^ Naresh K. Jena, ^c^ Sudip Chakraborty, ^c^ Jöns Hilborn,^a^ and Oommen P. Varghese^*,a^**

^a^Polymer Chemistry Division, Department of Chemistry-Ångström Laboratory, Uppsala University, Box 538, 751 21 Uppsala, Sweden

^b^BioMediTech, Bioengineering and Nanomedicine Lab, Biomaterials and Tissue Engineering group, Department of electronics and communications engineering, Tampere University of Technology, Tampere, 33720, Finland

^c^Condensed Matter Theory, Materials Theory Division, Department of Physics and Astronomy, Ångström Laboratory, Uppsala University, Box 516, 751 21 Uppsala

Table of Contents

Experimental method S3

Figure S1. Representative ^1^H NMR of oxime formation using (aminooxy)methane and a) 4-hydroxybenzaldehyde or b) acetone. S4

Figure S2. Representative examples pseudo first order reaction rate with 30 mM of (aminooxy)methane and 1 mM of acetone. S5

Figure S3. UV-vis spectra for reactions of 0.032 mM of 4-nitrobenzaldehyde and 1 mM (aminooxy)methane. S6

Figure S4. Representative examples of pseudo-first-order reaction rate of 0.032 mM of acetone/4-nitrobenzaldehyde and 1 mM (aminooxy)methane. S7

Figure S5. Cell surface versus intracellular oxime labeling in HCT116 cells. S7

Experimental Methods

**Pseudo-first-order oxime ligation kinetics analysed by ^1^H NMR**

All the reagents used in oxime ligation kinetics were dissolved in deuterated phosphate buffer (dPB, 10 mM), which was subsequently neutralized to pH 7.0 (pD 7.4) before use. The pD was calculated according to the equation pD = pH+0.4.

The pseudo-first-order reactions were performed using a 30-fold excess of aminooxy (30 mM) with respect to aldehyde/keto substrates (1 mM) in dPB. An appropriate catalyst amount was added and the pH value was adjusted to 7.0. ^1^H NMR spectra were recorded at appropriate time points and the extent of oxime ligation was quantified by integrating the methyl signal from free acetone at 2.24 ppm with the new methyl peak from the oxime product at 1.90 ppm (Figure. S1). When 4-hydroxybenzaldehyde was used as a substrate, the integral of the aldehyde signal at 9.72 ppm was integrated with the oxime proton at 8.20 ppm (Figure. S1). Pseudo-first-order reaction rate was calculated using equation (S1-S3). Representative examples of these pseudo-first-order reaction rates are presented in Figure. S2. Comparative pseudo first order rate kinetics was plotted in Figure. S2.

$\%Oxime=100\times\frac{A}{A+B}\%$ (S1)

where, *A* is the ^1^H NMR integration of peak from the methyl signal in acetoxime product at 1.90 ppm (acetone) or 4-hydroxy benzaloxime peak at 8.20 (4-hydroxybenzaldehyde), *B* is the integration value of the corresponding starting material peak at 2.24 ppm (methyl signal of acetone) or 9.72 (aldehyde proton signal of 4-hydroxybenzaldehyde)

$C_{-CHO}=\%Oxime\times1\times{10}^{-5}$ (S2)

$lnC_{-CHO}=-k_{obs}t$ (S3)

where *C_-CHO_* is a concentration of aldehyde (M) at time t (h), *k_obs_* is observed pseudo-first-order rate constant (h^-1^) and *c* is constant.

**Pseudo-first-order oxime ligation kinetics analysed by UV-Vis spectroscopy**

Samples were prepared in 3 ml standard quartz cuvette (path length 1 cm) by mixing the components. Phosphate buffer (10 mM, pH 7.4) containing 10 % (v/v) DMF and different concentrations of NaCl with or without aniline was used as a reference. 3 µl of 4-nitrobenzaldehyde stock solution (32 mM in DMF) was added to 2.982 ml of the above-mentioned mixture and UV-Vis absorbance (from 250 nm to 400 nm) was recorded. The reaction was initiated by the addition of 15 µl methoxyamine stock solution (200 mM in 10 mM phosphate buffer, neutralized to pH 7) and absorbance was recorded at specific time intervals. Time-dependent UV/Vis spectral changes are shown in Figure. S3. Absorbance at the 307 nm was plotted against time. Pseudo first order reaction rate was calculated using equation (S4 -S6). Representative examples of these time-dependent single-wavelength absorbance changes are presented in Figure. S3. Pseudo-first order rate kinetics was plotted in Figure. S4.

$\%Oxime=100\times\frac{A_{t}}{A_{max}}$ (S4)

$C_{-CHO}=\%Oxime\times3.2\times{10}^{-7}$ (S5)

$lnC_{-CHO}=-k_{obs}t$ (S6)

where, *A_t_* is an absorbance at time t, *A_max_* is maximum absorbance when *t*=∞,  *C_-CHO_* is a concentration of aldehyde (M) at time *t* (h), *k*_obs_ is pseudo first-order rate constant (h^-1^) and *c* is constant.


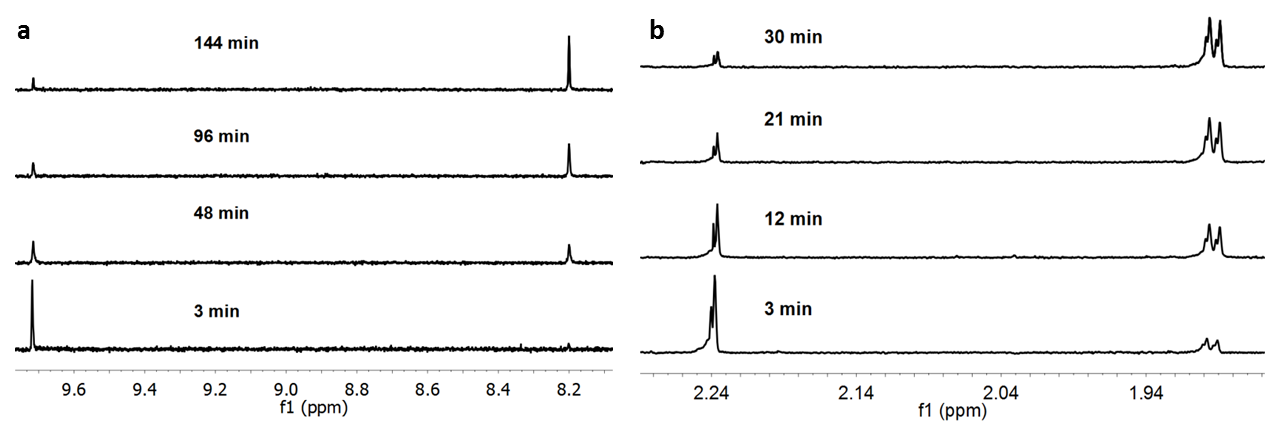


**Figure. S1** Representative ^1^H NMR of oxime formation using (aminooxy)methane and a) 4-hydroxybenzaldehyde or b) acetone.

**
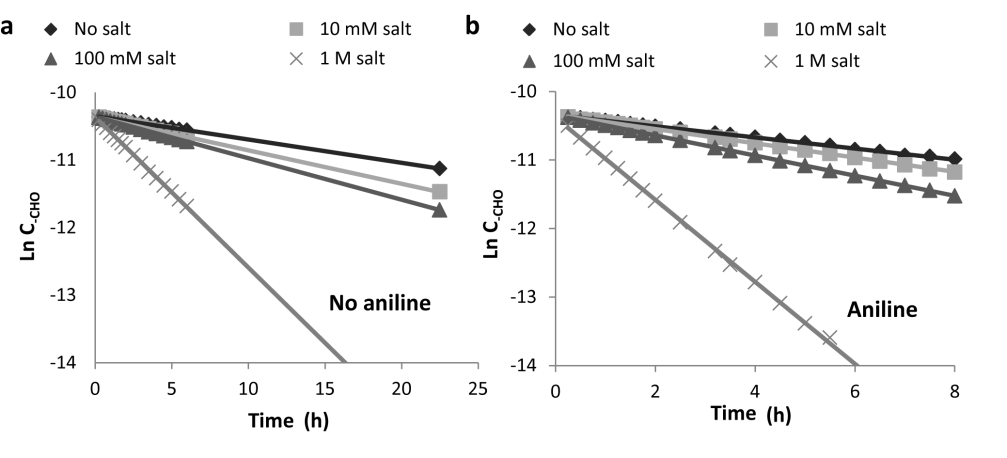
**

**Figure. S2** Representative examples pseudo first order reaction rate with 30 mM of (aminooxy)methane and 1 mM of acetone a) without aniline and b) with aniline.


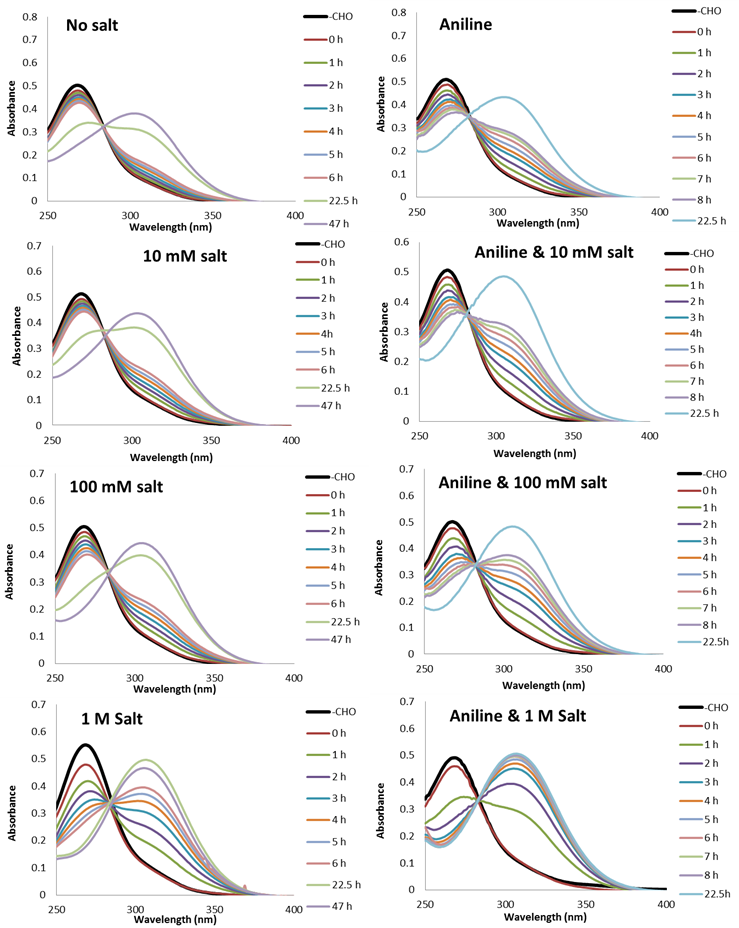


**Figure. S3** UV-vis spectra for reactions of 0.032 mM 4-nitrobenzaldehyde and 1 mM (aminooxy)methane with the different catalyst. Aniline concentration was maintained at 1 mM.


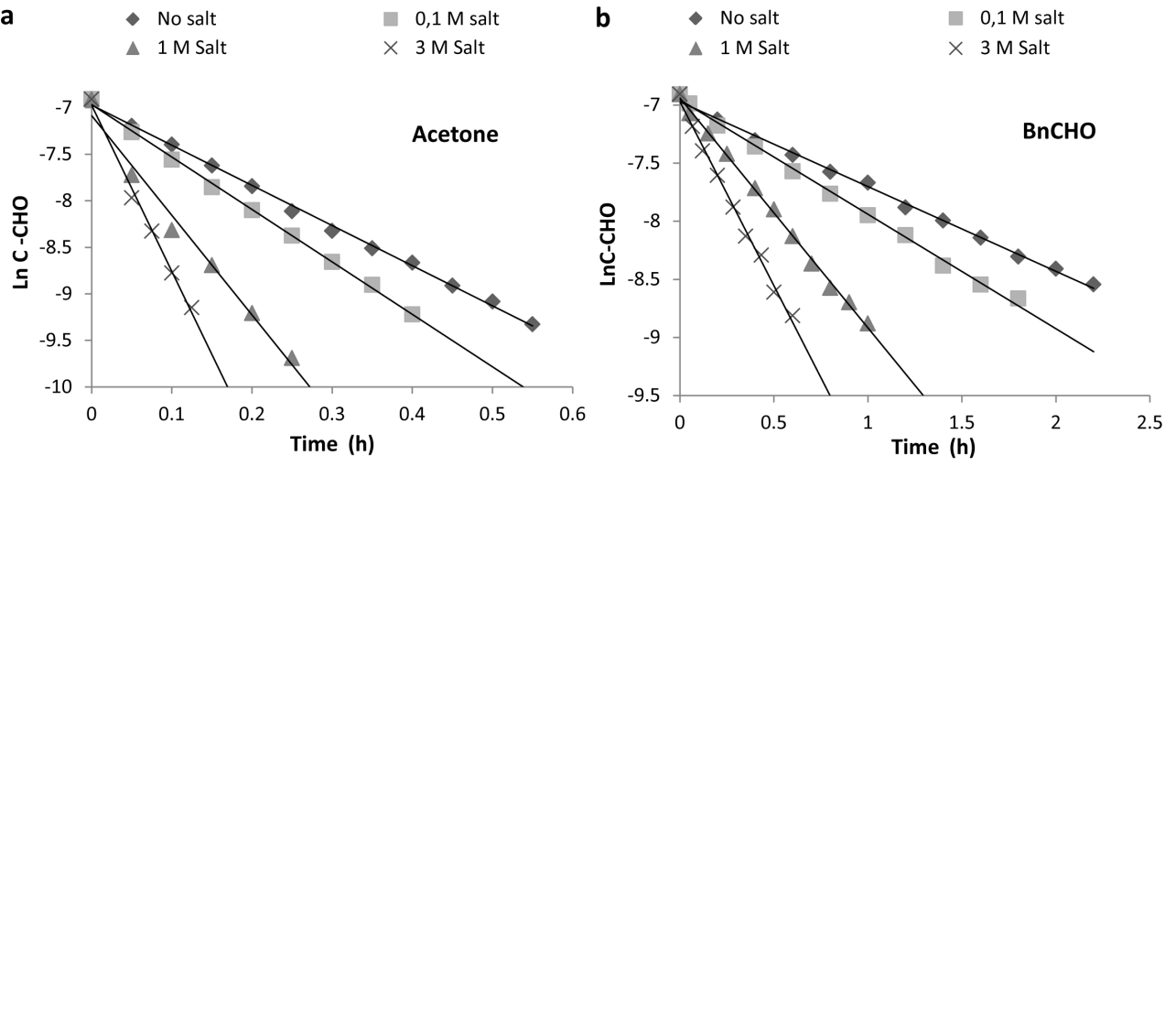


**Figure. S4** The representative examples pseudo first order reaction rate of a) acetone and b) 4-nitrobenzaldehyde.


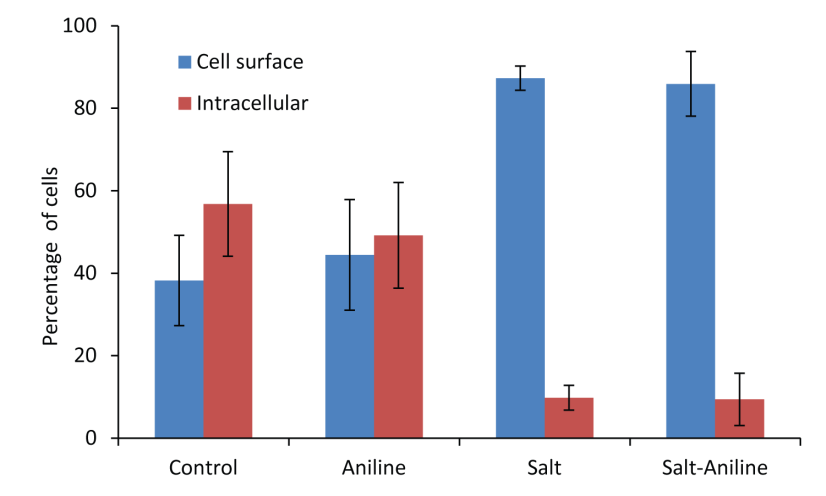


**Figure. S5** Cell surface versus intracellular oxime labelling of HCT116 cells. Analysis was performed with the images of 5000 cells obtained from Amnis flowsight.
